# Correction: Steppe et al. Bone Mass and Osteoblast Activity Are Sex-Dependent in Mice Lacking the Estrogen Receptor α in Chondrocytes and Osteoblast Progenitor Cells. *Int. J. Mol. Sci.* 2022, *23*, 2902

**DOI:** 10.3390/ijms23116020

**Published:** 2022-05-27

**Authors:** Lena Steppe, Jasmin Bülow, Jan Tuckermann, Anita Ignatius, Melanie Haffner-Luntzer

**Affiliations:** 1 Institute of Orthopedic Research and Biomechanics, University Medical Center Ulm, 89081 Ulm, Germany; lena.steppe@uni-ulm.de (L.S.); jasmin.buelow@uni-ulm.de (J.B.); anita.ignatius@uni-ulm.de (A.I.); 2 Institute of Comparative Molecular Endocrinology (CME), Ulm University, 89081 Ulm, Germany; jan.tuckermann@uni-ulm.de

The authors would like to make corrections to the reference citations in the original article [1]. The order of some reference citations has been adjusted correspondingly. The corrected version of this literature review addresses errors in the numbering of citations that were introduced during the proofreading stage of submission.

The authors apologize for any inconvenience caused and state that the scientific conclusions are unaffected. This correction was approved by the Academic Editor. The original publication has also been updated.
