# Supplementary material for: Bone Mass and Osteoblast Activity Are Sex-Dependent in Mice Lacking the Estrogen Receptor α in Chondrocytes and Osteoblast Progenitor Cells
Source: Int J Mol Sci. 2022 Mar 7;23(5):2902. doi: 10.3390/ijms23052902 (PMC8911122; doi:10.3390/ijms23052902)
Supplement: Supplementary file 1 [file ijms-23-02902-s001.zip › ijms-1621188-supplementary.pdf]

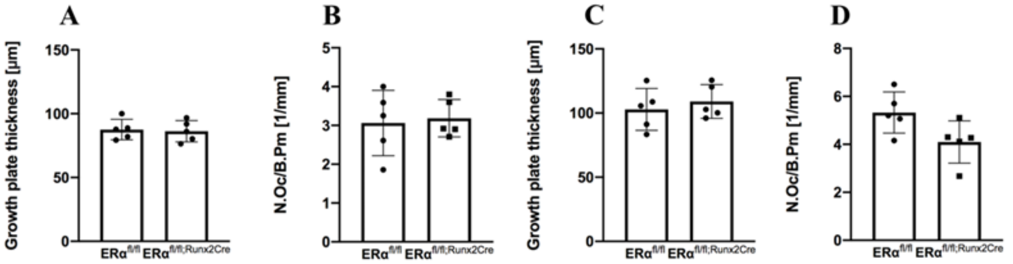

Figure S1. Femoral growth plate parameters of female and male ER$\alpha^{fl/fl}$ and ER$\alpha^{fl/fl;\ Runx2Cre}$ mice. (**A**) Growth plate thickness of female ER$\alpha^{fl/fl}$ and ER$\alpha^{fl/fl;\ Runx2Cre}$ mice, (**B**) osteoclast number per bone surface (OcS/B.Pm) of female mice at the growth plate, (**C**) growth plate thickness of male ER$\alpha^{fl/fl}$ and ER$\alpha^{fl/fl;\ Runx2Cre}$ mice, (**D**) osteoclast number per bone surface (OcS/B.Pm) of male mice at the growth plate. Data are shown as bar graphs (mean with SD), showing all data points. * Indicates significant effects with $p \leq 0.05$.

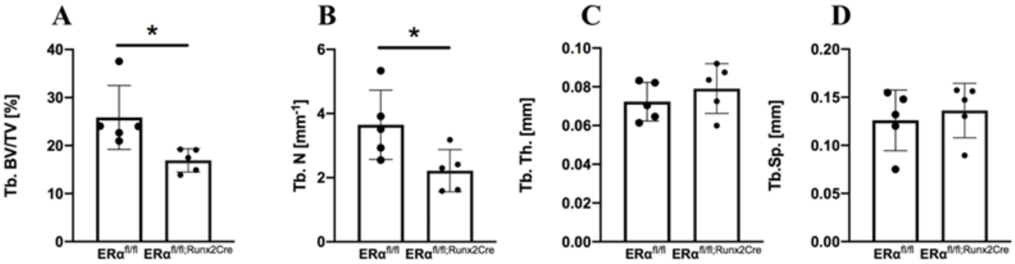

Figure S2. Bone microarchitecture parameters of the epiphyseal trabecular bone of the femur of female ER$\alpha^{fl/fl}$ and ER$\alpha^{fl/fl;\ Runx2Cre}$ mice. (**A**) Trabecular bone volume per tissue volume (Tb. BV/TV), (**B**) trabecular number (Tb. N), (**C**) trabecular thickness (Tb. Th.), (K) trabecular separation (Tb. Sp). Data are shown as bar graphs (mean with SD), showing all data points. * Indicates significant effects with $p \leq 0.05$.
